# *TaNAC020* homoeologous genes are associated with higher thousand kernel weight and kernel length in Chinese wheat

**DOI:** 10.3389/fgene.2022.956921

**Published:** 2022-08-26

**Authors:** Uzma Majeed, Jian Hou, Chenyang Hao, Xueyong Zhang

**Affiliations:** Key Laboratory of Crop Gene Resources and Germplasm Enhancement, Institute of Crop Sciences, Chinese Academy of Agricultural Sciences, Beijing, China

**Keywords:** thousand kernel weight, kernel length, KASP markers, *TaNAC020*, marker–trait association

## Abstract

NAC proteins constitute one of the largest plant-specific transcription factor (TF) families and play significant roles in plant growth and development. In the present study, three *TaNAC020* homoeologous genes located on chromosomes 7A, 7B, and 7D were isolated from wheat (*Triticum aestivum* L.). *TaNAC020s* were predominantly expressed in developing grains. The developed transgenic rice lines for *TaNAC020-B* showed higher starch density and lower amylose contents than those of the wild type (WT). Sequence polymorphism studies showed seven and eight SNPs in *TaNAC020-A/B*, making three and two haplotypes, respectively. No sequence polymorphism was identified in *TaNAC020-D*. Association analysis revealed that HAP-2 of *TaNAC020-A* and *TaNAC020-B* was the favored haplotype for higher thousand kernel weight and length. Geographic distribution and allelic frequency showed that our favored haplotype experienced strong selection in China, and likewise, diversity increased in *TaNAC020s* during wheat polyploidization. The results obtained in this study demonstrate that *TaNAC020s* positively influence starch synthesis and accumulation and are one of the key regulators of the kernel (seed) size and kernel number and have the potential for utilization in wheat breeding to improve grain yield. Molecular markers developed in this study stand instrumental in marker-assisted selection for genetic improvement and germplasm enhancement in wheat.

## Introduction

Common wheat is one of the most important cereal crops worldwide. Breeding for higher yield remains the major breeding objective due to the global population surge. In general, wheat yield is associated with kernel weight and kernel number per unit area (Kumbhar et al., 1982), while grain weight is the most important component of the grain yield. In China, merely a 1-g increase in thousand kernel weight (TKW) can result in almost 150 kg ha^−1^ increase in grain yield (Tian et al., 2006). Several genes influencing grain-related traits have been cloned, and their functional markers have been developed, such as *TaTGW6-*A1, *TaSnRK2.3*-1A/1B, *TaFlo2-*A1, *TaSnRK2.9-*5A, *TaZIM-*A1, and *TaCKX* (Hanif, et al., 2016; Miao et al., 2017; Sajjad et al., 2017, Ur Rehman et al., 2019; Liu et al., 2019; Shoaib, et al., 2020). However, the genes influencing kernel weight and the underlying molecular mechanism governing kernel weight in wheat still require more exploration. Hence, the continuous investigation on genes underpinning the kernel and direct yield–contributing traits is in progress.

Transcription factors (TFs) are involved in different developmental stages of crop plants. Although numerous NAC TFs have been functionally characterized in model plants such as *Oryza sativa* and *Arabidopsis thaliana*, the functions of most of the NAC family members are yet to be explored. About 263 NAC genes (http://planttfdb.gao-lab.org) have been identified in different crop plants, which show diverse expression patterns and are involved in multiple aspects of development and stress. It has been recently reported that three NAC TF–encoding genes—*OsNAC020*, *OsNAC023*, and *OsNAC026*—express mainly during seed development in rice (Mathew et al., 2016). The family name NAC is derived from the first three such transcription factors identified, namely, NAM (no apical meristem, *Petunia*), ATAF1-2 (*Arabidopsis* transcription activation factor) (Souer et al., 1996), and CUC2 (cup-shaped cotyledon, *Arabidopsis*), all of which have similar DNA binding domains (Aida et al., 1997; Souer et al., 1996) and comprise a large protein family. The NAC proteins are associated with biological functions, including embryonic, floral, and vegetative development (Souer et al., 1996), lateral root formation and auxin signaling (Xie et al., 2000), defense (Hegedus et al., 2003), and abiotic stress. In wheat, TF members are distributed across 40 families and 84 subfamilies. Likewise, other TFs in wheat, the plant-specific *NAC* family, also regulate several biological processes. Only a proportion of NAC proteins have been studied to date.

In crop genetics, the development and application of DNA markers have recently attained significant attention. SNPs are abundant in genomes and are regarded as the best markers for molecular breeding. Although the latest sophisticated genotyping platforms have considerably improved gene discovery studies by lowering the time and cost to do genotyping of large populations, still their usage in practical crop breeding is limited. The rate of historical genetic gains has illustrated that relying only on traditional breeding methods is not enough to fulfill the need for ever-increasing selection intensity and accuracy (Rutkoski et al., 2014). Two wheat varieties, Zhongmai 1062 and Jimai 23, have been developed using marker-assisted selection (MAS), which will be of great importance in achieving fast-forward genetic gain. Hence, genetic markers are ideal for the identification and selection of superior haplotypes in marker-assisted breeding for the improvement of crop plants.

Wheat genetic variations are determined by various factors such as polyploidization (Choulet et al., 2014), gene flow (Luo et al., 2007), the secondary center of origin (Zhou et al., 2018), domestication (Tanno and Willcox, 2006), and post–domestication selection (Cavanagh et al., 2013). During domestication and modern breeding, wheat experiences strong but artificial selection for the traits related to grain yield, quality, stress tolerance, and environmental adaptability (Cavanagh et al., 2013). Genes under artificial selection are usually associated with complex and vital agronomical traits (Zhou et al., 2018). Hence, information regarding genetic variability during crop improvement can give valued parameters for wheat breeding (Cavanagh et al., 2013).

NACs are mostly reported in response to abiotic and biotic stresses in plants. Increasing evidence reveals that NACs are also involved in various developmental processes in crop plants (Chen, 2017). In this study, a potential starch synthesis regulator in wheat, *TaNAC020*, was selected by co-expression analysis. The main objectives of this study were to 1) identify basic gene function by developing and studying phenotypes of transgenic rice, 2) to characterize sequence polymorphisms at *TaNAC020-A/B/D* loci on chromosome 7A/B/D, for the development of functional markers to characterize variation in the given genes, 3) evaluate the association of haplotypes with yield-contributing traits, and 4) to explore the geographic distribution of haplotypes among wheat accessions from China.

## Materials and methods

### Plant materials and trait phenotyping

Thirty-six highly diverse hexaploid wheat accessions (Supplementary Table S1) were used to identify the DNA sequence polymorphism of *TaNAC020-A/B/D*. Chinese wheat landraces (157 accessions) from the mini-core collection (Population-1) and 348 Chinese modern wheat (*Triticum aestivum* L.) cultivars (Population-2) (Supplementary Table S1) were used for association analysis between wheat germplasm and yield-related phenotypes. These populations were planted at Luyong, Henan province, China, during the 2002-03 and 2005-06 cropping seasons and at Shunyi, Beijing, China, in the 2010-11 cropping season. Field trials were conducted in a randomized complete block design (RCBD) with three replicates at all locations. Each plot consisted of three 2-m rows spaced 20 cm apart. The collection of agronomic traits data was followed as described by Zheng et al. (2014). Ten plants from the center of each plot were randomly sampled. Morphological traits included heading date (HD), maturity date (MD), spikelet number per spike (SNPS), thousand kernel weight (TKW), plant height (PH), spike length (SL), grain number per spike (GPS), effective tiller number (ETN), kernel length (KL), kernel width (KW), and kernel thickness (KT). Population-1 and population-2 were also investigated for allelic variation, selection pattern, and geographic distribution of identified SNP/haplotypes in China.

Sixty accessions of wheat and their wild relatives (diploid and tetraploid wheat) were used to assess the evolutionary behavior of the targeted genes (Supplementary Table S1). A set of nullisomic–tetrasomic and ditelosomic lines of “Chinese Spring” was employed to confirm the chromosomal location of *TaNAC020-A/B/D.*

### Isolation and sequencing of *TaNAC020s* and its basic function characterization through rice transformation

In co-expression analysis of TFs with starch synthesis genes in developing endosperms, we retrieved the sequence information of *TaNAC020s*. Full-length sequences of the three homoeologous genes were obtained by performing BLAST in the Chinese Spring Reference Genome 1.0. *TaNAC020-B* cDNA was amplified by C17720-2-1EF (F-‘TCTAGAATGGCAGACCACCTTCAAGTTC’) and C17720-2-984ER (R-‘GTCGACTCAGTAGTTCCACATGCCATCCA’) primers, and the target fragments were then ligated with *pEASY*-Blunt Cloning Vector. After sequencing, two endonuclease enzyme cutting sites for *Xba*l1 and *Sal*1 were added upstream and downstream of the ORF, respectively. A 30-bp MYC-tag sequence was inserted between the *Xbal*1 site and the first ATG of *TaNAC020-B*, followed by sub-cloning into the binary vector *pC2300-Actin1-ocs* cut with corresponding enzymes. The construct was then transferred into wild type rice (Kittakee) by the agrobacteria-mediated method after the transformation of the target gene into agrobacteria EHA105. Positive transgenic plants were initially screened by PCR, followed by sequencing for reconfirmation. T3 pure lines were used for phenotyping. The transgenic lines and the WT plants were planted at the Institute of Crop Sciences, Experimental Station in Beijing. Initially, rice seeds were germinated in Petri plates for 5 days, followed by transplantation into plastic tanks (L × W × H = 80 × 35 × 30 cm).

For morphological characterization of transgenic rice lines, T3 pure transgenic rice lines and WT plants were planted in an RCBD manner in triplicates. The morphological traits included PH, TKW, ETN, spike length (SL), and grains per spike (GPS). For the observation of starch granules, developing and mature grains of T3 transgenic rice seeds were dried completely under low pressure and cut across the short axis with a razor blade. The cross-sections were stammer coated with gold and observed by scanning electron microscopy (SEM) (HITACHI SU8000). Micrographs of each sample were taken with a magnification of ×600.

### RNA extraction and normalized fold expression

Various tissue samples (leaves, developing grains, stems, and roots) were collected at different developmental stages from Chinese Spring. Total RNA was extracted using RNAprep Pure Plant kit (Tiangen, Beijing, China). For each sample, approximately 2 µg of RNA was used for reverse transcription with a FastQuant RT Kit (Tiangen, Beijing, China). The qRT-PCR assays were carried out using SYBR Premix Ex Taq (Takara, Dalian, China) with Roche Light Cycler 96-well Real-Time PCR system (Roche, Switzerland). The transcript level of *TaNAC020* was also studied in T3 transgenic rice. Each experiment was triplicated using the SYBR Green PCR Master Mix Kit (Takara, Japan). The rice *Tubulin* and wheat *Actin* transcripts were used as an internal control to quantify the normalized fold transcript levels. The normalized fold expression levels were calculated using the 2^−ΔΔCT^ method (Livak and Schmittgen 2001). The primers used in this study are listed in Supplementary Table S2.

### Detection of *TaNAC020* diversity in wheat and related species

Genomic DNA from thirty-six highly diverse hexaploid wheat accessions was extracted from young leaves by the CTAB method. For the DNA polymorphism study, the wheat accessions from our previously reported studies were used (Su et al., 2011; Zhang et al., 2020). Three pairs of primers (TaNAC7A-1-F1/R1, TaNAC7B-1-F1/R1, and TaNAC7D-1-F1/R1) were selected to amplify *TaNAC020-A*, *TaNAC020-B*, and *TaNAC020-D* genomic sequence in wheat “A/B/D” genomes. Detailed information for PCR amplification is given in Supplementary Table S2. PCR products were checked on agarose gel (1%), and the desired bands were isolated and extracted using the Biomega gel purification kit, followed by cloning into the *pEASY*-Blunt vector. Ten positive clones for each sample were selected for sequencing on DNA Analyzer 3730xl (Applied Biosystems). To get a full-length desired sequence of the targeted genes, M13 forward and reverse primers and overlapping sequencing primers were used for sequence walking (Supplementary Table S2). The SeqMan program in the DNASTAR Lasergene software package was used to obtain the sequence of each clone by assembling it. The genomic origin of each sequence was confirmed by comparing it with the reference genome sequence obtained from URGI (https://urgi.versailles.inra.fr/blast/blast.php).

To study phylogeny, TaNAC protein members were aligned with NAC family members from other species. A neighbor-joining phylogenetic tree for *TaNAC020s* and NAC family members in wheat and other species was constructed, and the significance of the inferred relationships was determined by bootstrap analysis (1,000 replicates).

### Functional marker development

Two gel-free KASP markers (TaNAC020-A-KASP1 and TaNAC020-A-KASP2) were developed based on two selected polymorphism sites in the order of 612 nt (C/T) and 878 nt (T/G) of *TaNAC020-A* by following standard KASP guidelines (http://www.lgcgenomics.com). The allele-specific primers were developed with standard HEX and FAM tails with a targeted SNP at the 3′ end. The PCR reaction mixture consisted of 30 µL common primer (100 µM), 12 µL of each tailed primer (100 µM), and 46 µL ddH_2_O. The KASP assay was tested in ∼5 µL reaction mixture [2.4 µL 25 ng/μL DNA, 2.5 µL of 2 × KASP master mix, 0.06 µL primer mixture (all three primers), and 0.04 µL MgCl_2_].

A KASP marker (TaNAC-B-KASP) was developed based on the selected polymorphism site, 819 nt (C/T) for *TaNAC020-B*. The PCR reaction mixture and conditions are aforementioned. KASP primers information is given in Supplementary Table S3. Allelic discrimination plots were created using the QuantStudio™ 7 Flex (Applied Biosystems by Life Technologies), and data were visualized using the QuantStudio™ Real-Time PCR software v.1.3 (Applied Biosystems by Life Technologies).

### Marker trait association analysis

Descriptive statistics and estimates of variance were conducted using the SPSS system for Windows version 16.0 (http://www.-01.ibm.com/software/analytic/spss/). To determine phenotypic differences between genotypes, based on analysis of variance (one-way ANOVA), we used Student’s *t*-test at a significance level of *p* < 0.05. The polymorphic information content (PIC) and heterozygosity values (*H*_*e*_) were calculated using www.gene-calc.pl/pic website across entire wheat populations.

## Results

### Identification and genetic characterization of *TaNAC020s*

Co-expression pattern based on RNA sequencing results suggests that *TaNAC020* was expressive in 2, 11, 15, and 25 days after pollination (http://www.wheat-expression.com/) (Supplementary Figure S1). *TaNAC020-A*, *TaNAC020-B*, and *TaNAC020-D* encode proteins containing 204, 327, and 153 amino acid residues (AARs), respectively. The two homoeologous genes *TaNAC020-A/B* consist of a single intron and two exons, while *TaNAC020-D* endures intron-less structure with a single exon due to retro-transposition/duplication events that might have occurred during evolution. The genomic sequence lengths of *TaNAC020-A*, *TaNAC020-B*, and *TaNAC020-D* were 2009, 2027, and 2185 bps, respectively. Phylogenetic analysis revealed that *TaNAC020-A/B/D* has higher homology among them (Supplementary Figure S2A). Scan site analysis showed that *TaNAC020-A/B/D* have two regions, i.e., N-terminal region contains a NAM superfamily domain that is conserved across both monocots and dicots and acts as a DNA-binding domain, and the C-terminal transcription activation region which is remarkably divergent. The N-terminal region ranges from 17 to 143 AARs while having a diverse C-terminal region (Supplementary Figure S2B). The potential transmembrane-spanning region is absent.

By aligning to the genomic sequence of Chinese Spring (International Wheat Genome Sequencing Consortium, 2014), three homoeologous genes were mapped on chromosomes 7A, 7B, and 7D. Moreover, to determine the chromosomal location of *TaNAC020-A/B/D*, genome-specific primers (Supplementary Table S2) were designed. PCR amplification of the respective primers for each genome in diploid, tetraploid, nullisomic–tetrasomic, and ditelosomic lines revealed that *TaNAC020-A*, *TaNAC020-B*, and *TaNAC020-7D* were located on the short arm of the seventh chromosome of their respective genomes (Supplementary Figure S3).

### Overexpression of *TaNAC020-B* promotes starch synthesis and reduces amylose contents in rice

**FIGURE 1:**
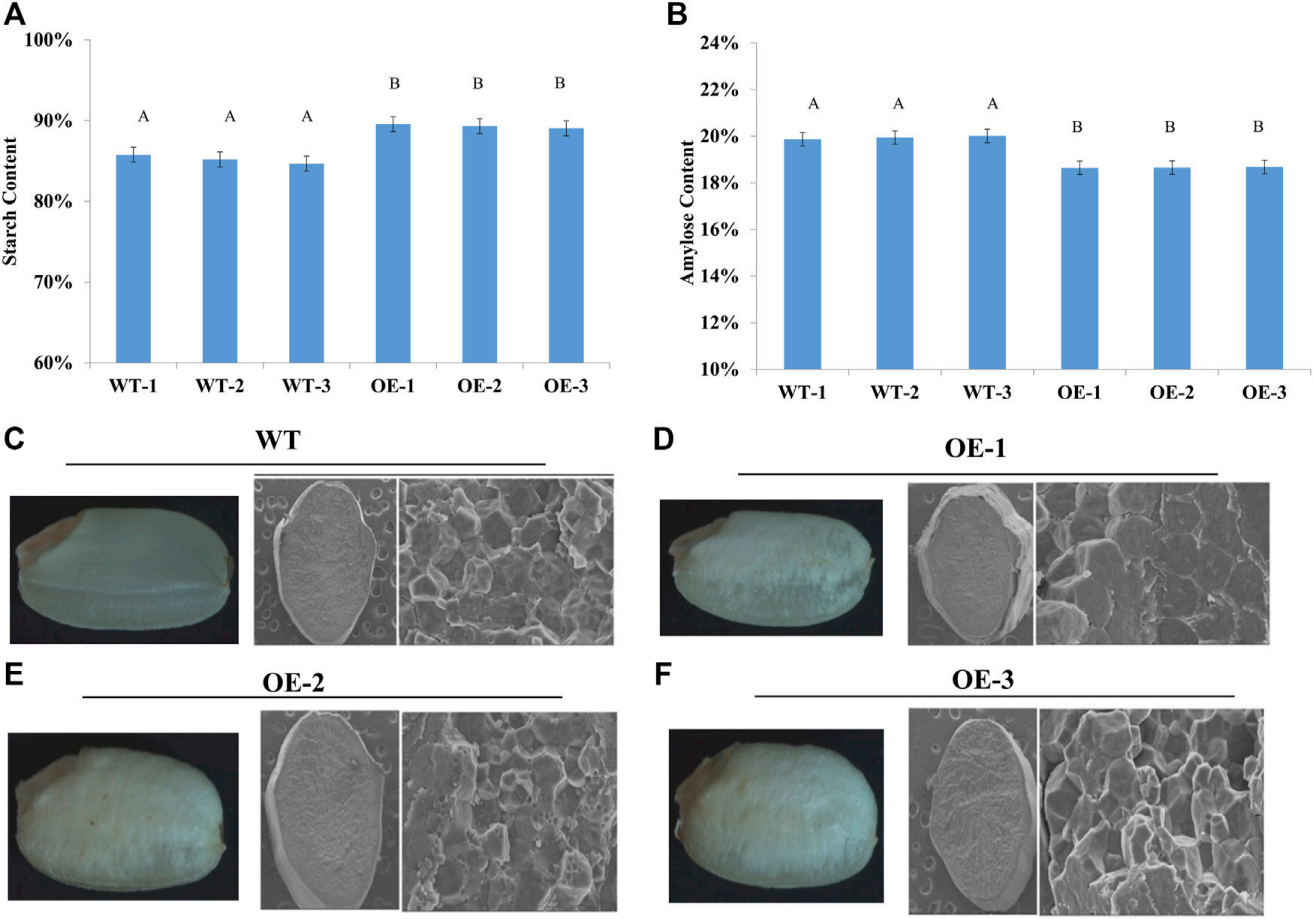
*TaNAC020-B* over-expressed rice heading color and kernel demonstrate their positive effects on starch synthesis and accumulation. **(A)** Starch content in wild type (WT) and over-expressing (OE) lines. **(B)** Amylose content in WT and OE lines. **(C–F)** Starch density and amylose content in WT and OE under scanning electron microscopy.

To test whether the starch granules in the endosperm were affected by *TaNAC020-B* overexpression in rice, transgenic rice plants (T3) were first screened on hygromycin plates, followed by reconfirmation with qRT-PCR. Three overexpressing (*TaNAC020-B*) transgenic rice lines were selected for morphological assays. The average data were recorded from six plants of each transgenic rice line and WT. The overexpressing rice lines showed higher TKW and KPS (Table 1). The over-expressed rice lines showed higher starch density and lower amylose contents than the wild type when seed cross-sections were examined under SEM. These results indicate that *TaNAC020-B* affects starch synthesis, amylose contents, kernel number, and TKW (Figure 1).

**TABLE 1:** Descriptive statistics based on mean and standard error for over-expressing transgenic rice lines to wild type. WT, wild type; O.E 1–O.E 3, transgenic rice lines; PH, plant height; KPS, kernel per spike; TKW, thousand kernel weight; TN, tiller number; SPL, spike length, NS, non-significant.

Mean and standard error					*p*-value		
Transgenic rice	WT rice	O.E 1	O.E 2	O.E 3	WT/O.E 1	WT/O.E 2	WT/O.E 3
PH	73.1 ± 1.6	74.4 ± 4.7	76.6 ± 2.2	74.3 ± 1.6	NS	NS	NS
KPS	65.7 ± 3.5	72.5 ± 1.7	76.5 ± 1.6	78.4 ± 2.3	0.015*	0.005**	0.004***
TKW	18.8 ± 0.6	19.8 ± 1.6	19 ± 1.2	18.9 ± 0.5	NS	NS	NS
TN	14.5 ± 1.7	20.3 ± 1.0	17.3 ± 1.8	17.8 ± 2.3	0.035	NS	NS
SPL	14.5 ± 0.7	14.7 ± 0.8	14.3 ± 0.9	14.6 ± 0.5	NS	NS	NS

### *TaNAC020s* are mainly expressed in developing grains

**FIGURE 2:**
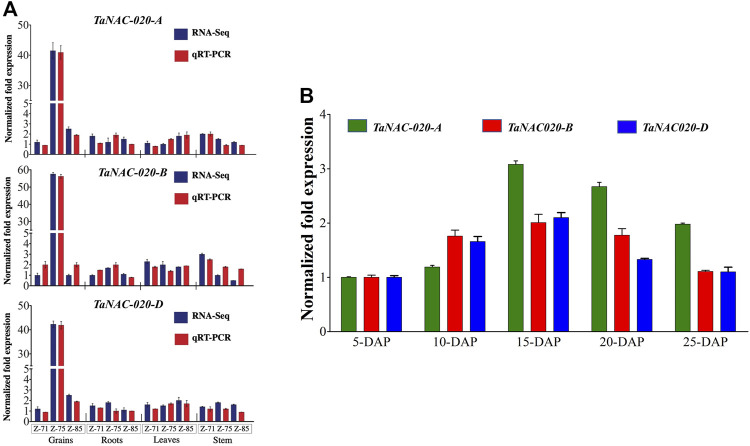
Gene expression pattern. **(A)** Tissue and stage-specific expression profile of candidate genes (Wheat-Exp database and qRT-PCR). **(B)** Normalized fold expression of *TaNAC020-*A/B/D at different stages of the day after pollination (DAP). Error bar denotes SE.

For the expression pattern of *TaNAC020-A/B/D*, qRT-PCR was employed in different tissues at different developmental stages of wheat. *TaNAC020-A/B/D* were highly expressed in grains, whereas substantially lower expressions were observed in leaves, stems, and roots (Figure 2). The higher expression was observed at the caryopsis stage (5 days post-anthesis) and peaked at the medium milking stage (15 days post-anthesis) followed by declined expressions at the soft dough stage (Figure 2).

### Association analyses and genetic effects of *TaNAC020s* haplotypes on yield contributing traits

**FIGURE 3:**
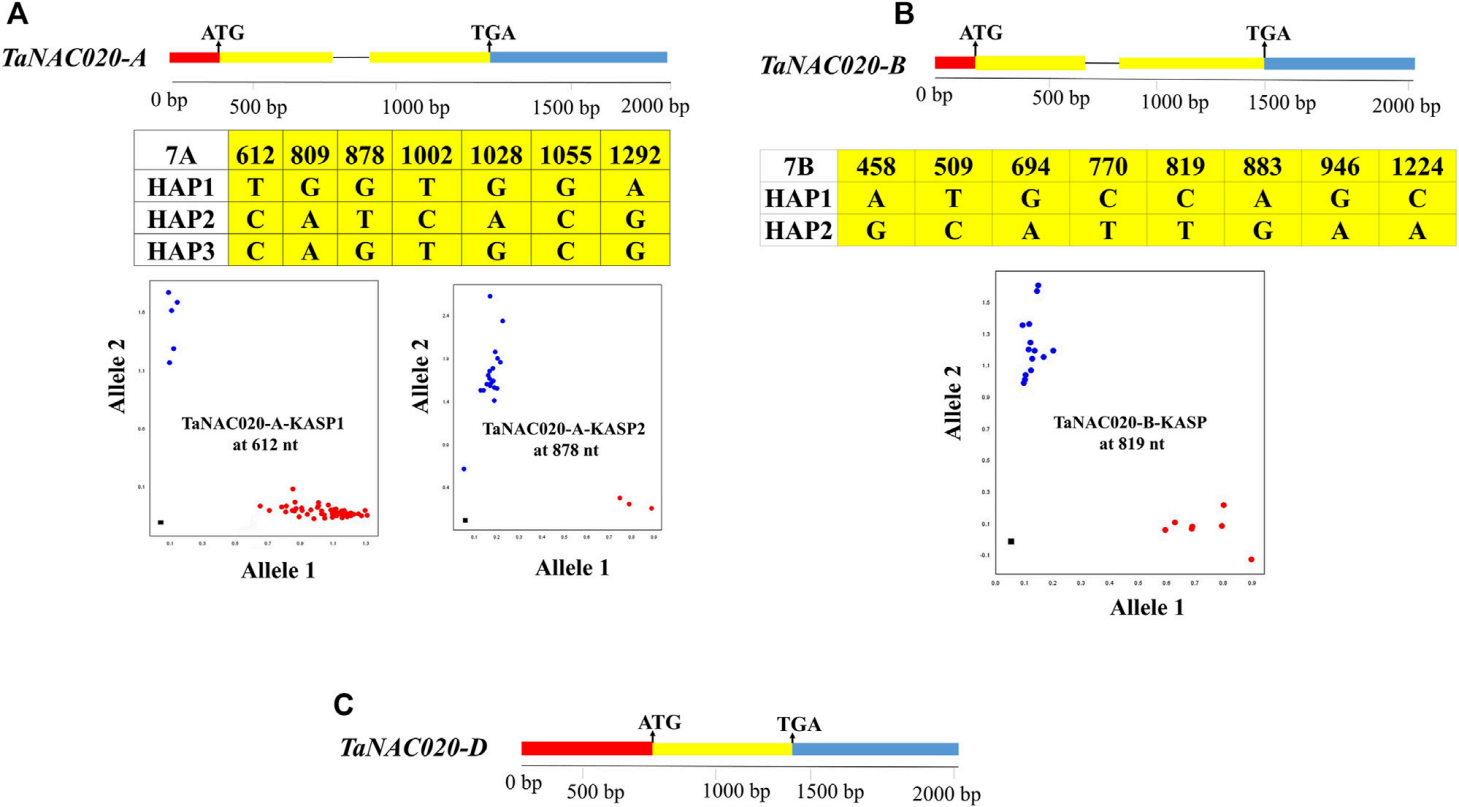
Molecular marker development for TaNAC020-A/B/D. **(A)** Gene structure, SNP sites, and KASP markers for *TaNAC020-A*. **(B)** Gene structure, SNP sites, and KASP marker for *TaNAC020-B*. **(C)** Gene structure of *TaNAC020-D*. Red = upstream of ATG, yellow = exon, black line = intron, and blue = downstream of TGA. Scatter plot for KASP assays showing clustering of accession on X-(HEX) and Y-(FAM) axes, and the black square represents non-template control.

Seven SNPs were identified in the target fragment of *TaNAC020-A*, eight SNPs were identified in the target fragment of *TaNAC020-B*, whereas no SNP was identified in the target fragment of *TaNAC020-D*. Two functional markers were developed to distinguish the haplotypes of *TaNAC020-A* based on the chosen SNP sites (Figure 3). Identified SNPs formed three haplotypes (HAP), namely, HAP-1 (TG), HAP-2 (CT), and HAP-3 (CG). To distinguish *TaNAC020-B* haplotypes, one KASP marker was developed based on the selected SNP site, resulting in the formation of two haplotypes (Figure 3).

**FIGURE 4:**
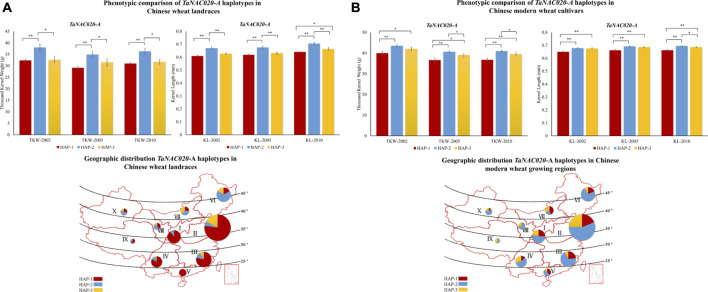
Phenotypic comparison of *TaNAC020-A* haplotypes and their distribution in 10 Chinese major wheat production zones. **(A)** Phenotypic comparison and geographic distribution of haplotypes in Chinese wheat landraces. **(B)** Phenotypic comparison and geographic distribution of haplotypes in Chinese modern wheat cultivars. *p < 0.05, **p < 0.01, error bar denotes SE. The size of the pie chart is directly proportional to the number of accessions from the agroecological zone.

**FIGURE 5:**
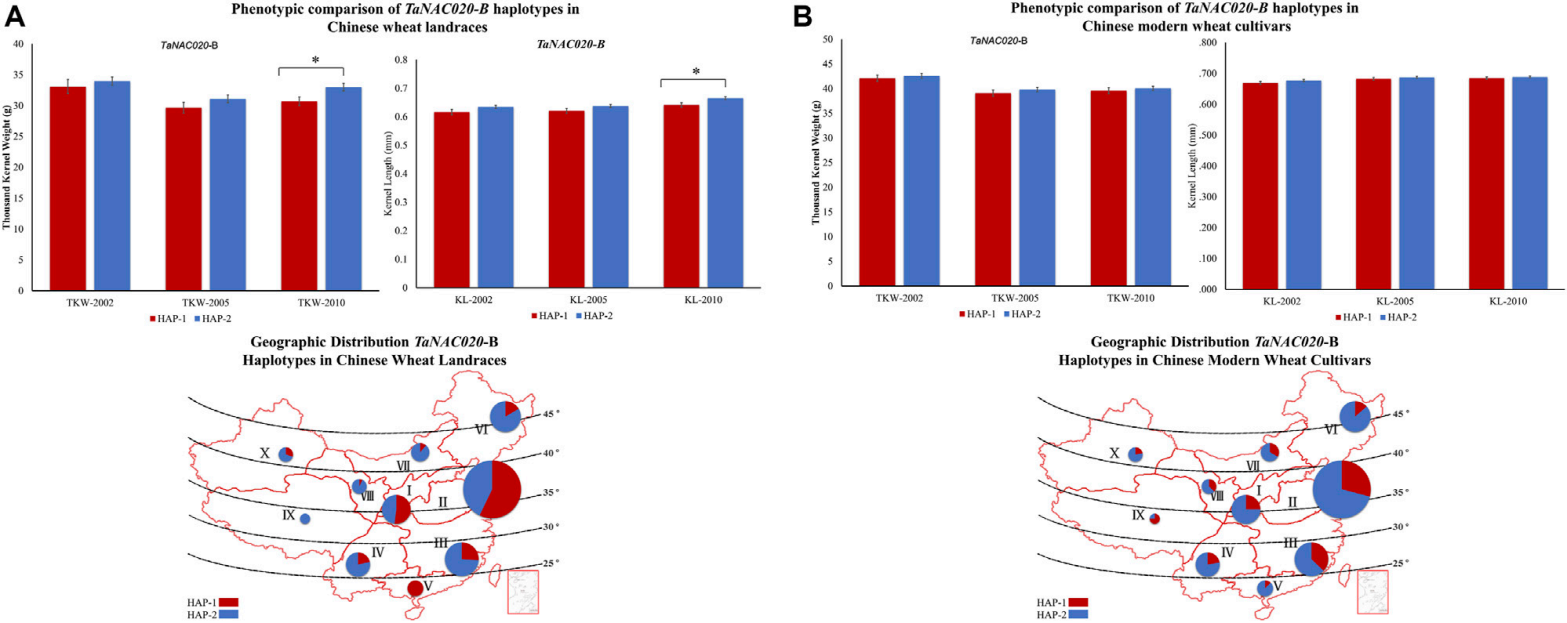
Phenotypic comparison of TaNAC020-B haplotypes and their distribution in 10 Chinese major wheat production zones. **(A)** Phenotypic comparison and geographic distribution of haplotypes in Chinese wheat landraces. **(B)** Phenotypic comparison and geographic distribution of haplotypes in Chinese modern wheat cultivars. *p < 0.05, **p < 0.01, error bar denotes SE. The size of the pie chart is directly proportional to the number of accessions from the agroecological zone.

Accessions containing HAP*-*2 of both *TaNAC020-A* and *TaNAC020-B* showed higher TKW and KL in all environments and both wheat populations (Figures 4, 5). These results suggest that both haplotypes might be superior haplotypes for higher TKW and KL in the studied Chinese wheat germplasm. To investigate the evolutionary history of *TaNAC020s*, we analyzed the given genes in wheat progenitors. An increasing trend in the values of PIC and *H*_*e*_ were observed from tetraploid to hexaploid wheat (Supplementary Table S4).

### Geographic distribution of *TaNAC020s* haplotypes in china

Variations in favored alleles tend to accumulate during the process of artificial selection. To evaluate comprehensively and systematically the distribution of all *TaNAC020s* haplotypes and to determine whether the favored haplotypes were selected in wheat breeding, we investigated the geographic distribution in China. China has three major wheat-growing regions which are further divided into 10 agroecological zones. The frequencies of favored haplotypes of *TaNAC020-A/B* were higher in all major wheat-growing regions in modern Chinese wheat cultivars than in landraces illustrating the strong positive selection of favored haplotypes during wheat breeding (Figures 4, 5). From landraces to modern cultivars, the frequency of favored haplotype of *TaNAC020-A* increased remarkably from 10.5 to 53.7% in zone I and 5.7–56.4% in zone II. These results indicated that the favored haplotype experienced strong positive selection in Chinese wheat breeding progress, and it positively regulated TKW and KL. For *TaNAC020-B*, the frequencies of favored haplotype for TKW and KL also increased from 48 to 75% in zone I and 42.8–70.9% in zone II (Figure 5). As TKW and KL are direct yield contributing traits, simultaneous selection of these traits for yield improvement led to their similar selection frequencies.

## Discussion

Plant-specific NAC transcription factors have been reported to play a diverse role in developmental processes in different crop plants, such as cell wall biosynthesis, leaf senescence, root development, seed development, stay-green, and nutrient remobilization (Christiansen et al., 2011; Zhang et al., 2019). In this study, we identified *TaNAC020s* in common wheat. N-terminal myristoylation is vital for protein function in mediation membrane association in plant responses to external factors (Podell and Gribskov 2004). The N-terminal myristoylation region has been identified in this study, and in plants, these regions are involved in protein–protein interaction and respond to abiotic stresses. In this study, *TaNAC020* expression patterns during wheat grain development were studied from 5-DAP to 25-DAP. The expression peaked at 15-DAP, followed by downregulation, suggesting that NAC transcription factors influence processes that promote the synthesis of photosynthetic machinery instead of degradation since NAC is reduced following leaf senescence. Expression analysis also indicated that *TaNAC020s* have a grain-specific expression pattern. *TaNAC020* homoeologous genes were highly expressed in grains at the medium milking stage, the period during which starch is being synthesized in the endosperm. Amyloplast synthesis is completed in 15–20 days post-anthesis, but starch synthesis can be detected even at 35–40 days post-anthesis in the endosperm in major wheat production regions in China. The expression of *TaNAC020-A* and *TaNAC020-B* positively correlates with starch synthesis–related genes. Moreover, *TaNAC020s* have higher sequence similarities (>98%) and play a similar function. In this study, overexpression of *TaNAC020-B* in T_3_ rice lines has shown higher TKW and KN than the wild type.

### Sub-function differentiation of three homoeologous (*TaNAC020-A/B/D*) in wheat

Plant breeding coupled with phenotypic and genotypic selections will accelerate the future breeding process. Haplotype blocks combining two or more SNPs in strong linkage disequilibrium are more explanatory than bi-allelic SNPs (Stephens et al., 2001). Brinton et al., 2020 have also supported the haplotype-led approach as an efficient tool for precise wheat breeding. Beyond bi-allelic SNP variations, the haplotype data could capture associations that evade identification by solitary SNPs (Lorenz et al., 2010). Haplotype-based analyses are still rare in wheat, with few exceptions (Li et al., 2016; Miao et al., 2017; Ur Rehman et al., 2019). Identification of haplotypes with improved phenotypes could accelerate genetic gain in crop improvement. Haplotypes can also capture epistatic interactions between SNPs. Hence, haplotype-based approaches could boost prediction accuracies (Bevan et al., 2017). Considering the genetic variations, most of the variabilities in complex traits, that is, grain yield, are influenced by polymorphisms in the regulatory gene rather than in the structural gene (Pflieger et al., 2001). Sequence polymorphisms in the genes encoding transcription factors are considered to be an important resource for developing functional markers (FMs). The challenge is, therefore, to link these sequence polymorphisms with agronomic traits. Thus, the significance of the contrast between *TaNAC020-A/B* haplotypes and the agronomic data obtained in three growing seasons was calculated.

Although domestication and modern-day breeding contribute to the development of various ecotypes and cultivars, genetic variation of genes governing phenotypic traits has also increased. We cloned and characterized *TaNAC020-A/B/D* in wheat. SNPs were identified in *TaNAC020-A* and *TaNAC020-B*, while no sequence polymorphism was identified in *TaNAC-D*. *Aegilops* L. genus, the “B” genome donor of *Triticum aestivum*, often supports a cross-pollination mechanism (Yuan et al., 2017) that might be the cause of a higher number of polymorphisms. It has been well documented that wheat “D” genome is more conserved when compared to “A” and “B” genomes because “A” and “B” sub-genomes of wheat experienced a transition from diploid to tetraploid (Cavanagh et al., 2013; Li et al., 2016; Miao et al., 2017; Ur Rehman et al., 2019; Hao et al., 2020). Wheat “A” genome possesses more agronomic trait-related genes than the “B” genome, and the selection pressure to “A” genome is stronger than the “B” genome (Peng et al., 2003). The probable reasons for no sequence polymorphism in *TaNAC020-D* are 1) *TaNAC020-D* might become more fixed because of artificial selection pressure, 2) the genetic diversity of accessions examined here is still not big enough to explore more SNPs, 3) the self-pollinating nature of wheat “D” genome donor ancestors.

### Prospective molecular markers in wheat breeding

SNPs are abundant in genomes and are regarded as the best markers. At the same time, FMs derived from polymorphic sites contained by genes causally involved in agronomic trait variation can be utilized to fix alleles in numerous genetic backgrounds deprived of additional calibration (Andersen and Lubberstedt 2003; Bagge et al., 2007). However, it is challenging to develop FMs in wheat due to the allo-hexaploidy nature (Bagge et al., 2007). At least three copies of ∼75% of wheat genes are present on homoeologous chromosomes having identical nucleotide sequences, which causes further difficulties in characterizing them separately (Hao et al., 2020). For a better understanding, the pattern of sequence polymorphism permits plant breeders to identify new alleles for breeding. Therefore, the present study investigated the genetic diversity for *TaNAC020s* in the Chinese wheat agroecological zone to facilitate wheat breeding and the use of developed molecular markers in marker-assisted breeding.

In this study, we developed SNP-based (KASP) markers. These markers are suitable and reliable for characterizing *TaNAC020* haplotypes in wheat germplasms in a simple, quick, and cost-effective (30–45% less expensive than other available conventional methods) manner. These are helpful in marker-assisted breeding aimed at grain yield (Rasheed et al., 2016). However, more work is required to exploit the aforementioned probabilities in a more diverse wheat germplasm. Also, bioinformatics algorithms and tools could be used to further explore the current polymorphism data for the desired yield-related traits under biotic and abiotic stress environments.

### Agronomic and physiological traits associated with *TaNAC020s* haplotypes

TKW and KL are among the most important agronomic traits and are often under high selection pressure. In wheat, kernel weight and kernel size have a positive correlation between them (Ali et al., 2020). High heritability values of TKW have proved that it is phenotypically a stable yield parameter, which continuously attracts wheat breeder attention because TKW has continuously improved during evolution, and this improvement may be due to the accumulation of preferred haplotypes associated with this trait. Likewise, accessions containing favored haplotypes of *TaNAC020-A* and *TaNAC020-B* are associated with higher TKW and KL. The aforementioned outcomes indicate that *TaNAC020* plays its role in governing TKW and KL in wheat.

Our haplotype association analysis also illustrated that HAP-2 of *TaNAC020-A* was significantly associated with shorter PH. In addition, PH decreased while TKW increased continuously over decades of Chinese wheat breeding history (Ur Rehman et al., 2019). PH is a trait easy to measure and remains constant after anthesis. Plant breeders prefer wheat PH ranging between 85 and 100 cm (Cui et al., 2011; Zhang et al., 2011). TKW and KN association is population dependent; a positive correlation has been reported between the aforementioned traits in Chinese modern wheat cultivars (Zhang et al., 2012). Since 1950, wheat varieties in China have changed 4 to 6 times, with approximately a 10% yield increment in each cycle (Zheng et al., 2014). In the 1960s, yield increase in wheat arose from the introduction and wide usage of *Rht1* and *Rht2* (Hedden, 2003). The yield increase in China has largely depended on higher TKW with semi-dwarf PH. In this study, HAP-1 of *TaNAC020-A* was the predominant haplotype (67.52%) in Chinese wheat landraces, whereas its frequency decreased to 18.97% in Chinese modern wheat cultivars. The frequency of HAP-2 increased remarkably from Chinese wheat landraces to Chinese modern wheat cultivars, suggesting a positive selection for HAP-2 in the regions during modern wheat breeding. Thus, trends for haplotype selection changed from HAP-1 in landraces to HAP-2 in modern Chinese cultivars, indicating the positive selection for HAP-2 having plants with reduced PH and higher TKW in modern wheat breeding practices. For *TaNAC020-B*–favored haplotypes, HAP-2 was found prominent in surplus water areas and highly associated with TKW. Thus, a cumulative effect of these superior haplotypes for TKW and KL caused a balancing effect in their selection frequencies from Chinese wheat landraces to modern wheat cultivars. Advancements in agro-industries in China strongly impacted grain yield. Small grains were replaced with larger grain sizes by intensive breeding with advancing technologies of milling.

The wheat germplasm panel selected in this study has high *H*_*e*_ and PIC, indicating higher polymorphism (Supplementary Table S4). Furthermore, *TaNAC020-B* had higher *H*_*e*_ and PIC, followed by *TaNAC020-A*, consistent with previous reports regarding higher polymorphism in the wheat B genome (Yuan et al., 2017).

Functional markers of several yield-related genes have been reported. Pyramiding preferred alleles of these genes with MAS will be instrumental in wheat breeding by enhancing additive genetic variation. Plant breeding through phenotypic selection is a time-consuming and relatively inefficient process (Gedye et al., 2012). Recently, remarkable progress has been made in developing FMs for marker-assisted selection in wheat for important agronomic traits, such as TKW, KN, and PH (Zheng et al., 2014; Hanif et al., 2016; Li et al., 2016; Miao et al., 2017; Sajjad et al., 2017; Ur Rehman et al., 2019; Shoaib et al., 2020; Ur Rehman et al., 2021). This study provides KASP FMs for *TaNAC020-A/B* genes with potential application in wheat breeding.

## Conclusion

The results indicate that *TaNAC020* exhibits multiple functions for grain development with increased kernel length and TKW. We also established high throughput and cost-effective molecular markers for *TaNAC020*-*A/B* genes. The genes identified here and molecular markers developed to identify haplotypes are useful for marker-assisted breeding for kernel length and high TKW. These markers can be used alone or in combination with other functional markers. Thus, *TaNAC020s* have the potential to be used in wheat yield improvement programs due to their key importance in plant growth and development.

## Data Availability

The original contributions presented in the study are publicly available. This data can be found here: https://www.ncbi.nlm.nih.gov/genbank/ ON960301, ON960302 and ON960303.
